# Supplementary material for: Social determinants of self-medication with leftover antibiotics in Lebanese households: A cross-sectional study
Source: PLoS One. 2025 Sep 25;20(9):e0333377. doi: 10.1371/journal.pone.0333377 (PMC12463266; doi:10.1371/journal.pone.0333377)
Supplement: S2 File — (DOCX) [file pone.0333377.s002.docx]

**QUESTIONNAIRE:**

Dear Participant,

Thank you for your cooperation in our research study conducted by the Lebanese University, School of Pharmacy, Master's Program. We aim to assess the influence of the social determinants of health (SDOH) on self-medication with antibiotic leftovers among the general population in Lebanon.

The SDOH are non-medical factors that affect health outcomes, including education, financial situation, social support, and living conditions. This study explores how these factors impact self-medication with antibiotic leftovers, which is defined as taking an antibiotic available at your home or borrowed from other persons without consulting a doctor, to treat or prevent an infection.

This survey will take approximately 15 minutes to complete. Please read each statement carefully and select the answer that best describes you. Rest assured that all information will remain confidential and will only be used for research purposes.

Your participation is voluntary, and you may withdraw from the survey at any time. While there are no direct benefits to participation, your input will contribute to our understanding of the SDH drivers of self-medication with antibiotic leftovers in Lebanon.

By proceeding with the survey, you indicate your consent to participate in this study.

Thank you for your valuable contribution to this research.

The Research Team

**Please check all the below statements to proceed to the survey.**

- I have read and understood the above information.
- I understand that my participation is voluntary.
- I understand that my data will be kept confidential.
- I agree to participate in this study.
- I pledge to submit true and reliable information.

**Did you ever use antibiotics leftover without consulting a physician to treat or prevent an infection?**

- No
- Yes

**Part I. Socio-demographic characteristics**

**How old are you (years)?**

**What is your marital status?**

- Single
- Widowed
- Divorced
- Married

**Do you have children?**

- No
- Yes

**What is your gender?**

- Male
- Female

**What is the highest level of education you have completed?**

- Less than high school
- High school diploma or equivalent
- University degree (completed or in progress)
- Master’s, PharmD, or Ph.D.

**What is the type of education?**

- Health Education
- Non-health education

**What is your area of residence?**

- Beirut
- North
- Mount Lebanon
- South
- Akkar
- Baalbek/Hermel
- Beqaa
- Nabatiyeh

**How do you describe your area of living?**

- Urban (cities, towns, suburbs)
- Rural (village)

**What is the distance between your home and the nearest pharmacy?**

- <1 km
- 1-5 km
- 5-10 km
- More than 10 km

**Do you suffer from any chronic illness?**

- No
- Yes

**What is the number of people living in the house?**

**What is the number of rooms at home, excluding the kitchen and bathroom?**

**Part II: Practice of self-medication**

**Where did you get the medications used without a prescription?** (Check all that applies)

- Unused antibiotics/leftover antibiotics from previous prescriptions at your home
- Family/Relative
- Neighbor
- Friends

**Indicate the reasons that encourage you to self-medicate** (Yes/no)

- Prior experience of the infection
- The infection treated was minor (not serious)
- Ease of antibiotics availability
- Convenience
- Prior experience with the drug
- Sufficient knowledge about the drug
- Inadequate time to attend the doctor’s office
- To save time
- To save money
- Shortage of money
- To get a quick fix and relief
- Healthcare access was not available
- Better privacy
- Lack of trust in the physician
- Do not like visiting the physician and getting check-ups
- It is not necessary to go to the physician for common illnesses

**Indicate the reasons that prevent you from self-medicating with antibiotic leftovers** (Yes/no)

- Lack of knowledge on dosage and frequency of doses
- The physician diagnoses the illness better
- Medical consultation is always better
- Risk of adverse events and side effects reactions
- There is no need
- Other safety concerns

**Part III: Social Determinants of Health:**

1. **Financial Resource Strain (The SES scale)**

**What do you feel is the level of your financial stress today?** (1= Overwhelming stress and 10=No stress at all)

**How satisfied are you with your present financial situation? (**1= Dissatisfied and 10= Satisfied)

**How do you feel about your current financial situation?** (1= I feel overwhelmed and 10=I feel comfortable)

**How often do you worry about being able to meet normal monthly living expenses?** (1= Worry all the time and 10=Never worry)

**How confident are you that you could find the money to pay for a financial emergency that costs about 5,000,000 L.L?** (1= No confidence and 10= High confidence)

**How often does this happen to you? You want to go out to eat, go to a movie, or do something else and not go because you can’t afford to.** (1= All the time and 10=Never)

**How frequently do you find yourself just getting by financially and living paycheck to paycheck?** (1= All the time and 10=Never)

**How stressed do you feel about your finances in general?** (1= Overwhelming stress and 10= No stress at all)

1. **Discrimination in medical settings (The DMS scale)**

**When you attend a medical setting, such as a hospital, clinic, or primary healthcare setting, how do you describe the physician or nurse's behavior?**

(Never, Rarely, Sometimes, Most of the time, Always)

- You are treated with less courtesy than other people
- You are treated with less respect than other people
- You receive poorer service than others
- A physician or nurse acts as if he or she thinks you are not smart
- A physician or nurse acts as if he or she is afraid of you
- A physician or nurse acts as if he or she is better than you
- You feel like a physician or nurse is not listening to what you are saying

1. **The political and socio-economic problems in Lebanon**

**How did the ongoing political conflict (War against GAZA) influence your decision to self-medicate?** (1: No influence at all, 5: Very strong influence)

**How did the unstable political situation in Lebanon influence your decision to self-medicate?** (1: No influence at all, 5: Very strong influence)

**How did the economic crisis in Lebanon influence your decision to self-medicate?** (1: No influence at all, 5: Very strong influence)

**How did the fear of an antibiotic shortage in Lebanon influence your decision to self-medicate?** (1: No influence at all, 5: Very strong influence)

1. **The trusted source of information**

**What was the trusted source of information about medications you used without a physician's prescription?** (yes/no)

- Pharmacist
- Previous experience with a similar disease
- Opinion from a family member/ friend
- Academic knowledge
- Internet (Google, PubMed)
- Reading materials (Newspaper/magazines/leaflets)
- Social media (Instagram, Facebook)
- Mass media advertisements (TV, radio)
- Artificial Intelligence (ChatGPT)

1. **Level of health literacy:**
   1. **Knowledge about antibiotic use**

**Do you agree with the following sentence?** (Strongly disagree 🡪 Strongly agree)

- Antibiotics can cause an allergic reaction
- Antibiotics can cause vomiting and nausea
- Antibiotics can cause diarrhea
- Antibiotics can cause abdominal pain
- Antibiotics can cause skin reactions (rashes/ulcers)
- Antibiotics can cause liver toxicity
- Antibiotics can cause kidney toxicity
- If you get side effects during a course of antibiotic treatment, you should see your physician immediately
- If you have a history of side effects or an allergy to antibiotics, you should inform your doctor/pharmacist
- Antibiotics can cause an imbalance in the body’s bacterial flora (good bacteria)
- It is okay to use leftover antibiotics from previous treatments (reverse coding)
- It’s okay to use antibiotics that were given to a friend or family member, as long as they were used to treat the same illness (reverse coding)
- It’s okay to save antibiotics for later use (reverse coding)
- It is okay to buy antibiotics without a prescription (reverse coding)
- I should stop taking antibiotics used for my treatment when I feel better (reverse coding)
- Body aches can be treated with antibiotics (reverse coding)
- Headaches can be treated with antibiotics (reverse coding)
  1. **Knowledge about antibiotic resistance:**

**Do you agree with the following sentence?** (Strongly disagree 🡪 Strongly agree)

- Many infections are becoming increasingly resistant to treatment by antibiotics
- Antibiotic resistance is an issue that could affect me and the future generation
- Antibiotic resistance is an issue in other countries but not in my country (reverse coding)
- Bacteria that are resistant to antibiotics can be spread from person to person
- You can be a carrier of resistant bacteria and pass them to your friends and/or family members
- If you become sick and your bacteria are resistant to your prescribed antibiotic, your illness could last longer
- Taking antibiotics unnecessarily may contribute to the development of antibiotic resistance
- The use of antibiotics among animals can reduce the effect of antibiotics on humans
- Animals can act as carriers of the resistant bacteria and pass them to humans
  1. **Knowledge of risks associated with Leftover Antibiotic use:**

**Do you agree with the following sentence?** (Strongly disagree 🡪 Strongly agree)

- Self-medication is a safe practice (reverse coding)
- There is a failure to recognize or report adverse drug reactions due to self-medication.
- There is a risk of double medication (with two different brands of the same drug) or harmful interaction.
- Self-medication may lead to inadequate or excessive dosage.
- Self-medication may cause the use of drugs in self-limiting conditions.
- Self-medication may lead to unnecessary under-use or prolonged use of drugs.
- Stopping prematurely antibiotics therapy
- Incorrect self-diagnosis (e.g., severe diseases remain unnoticed)
- Incorrect choice of therapy
- Failure to recognize or self-diagnose contraindications, interactions, warnings, precautions, etc...
- Risk of dependence and abuse
- Wastage of money if actual disease is not identified
