# Supplementary material for: Social determinants of self-medication with leftover antibiotics in Lebanese households: A cross-sectional study
Source: PLoS One. 2025 Sep 25;20(9):e0333377. doi: 10.1371/journal.pone.0333377 (PMC12463266; doi:10.1371/journal.pone.0333377)
Supplement: Attachment — Submitted filename: PONE-D-25-10954_reviewer.pdf [file pone.0333377.s003.pdf]

PLOS ONE

Social Determinants of Self-Medication with Leftover Antibiotics in Lebanese Households: A Cross-Sectional Study.

--Manuscript Draft--

**Manuscript Number:**	PONE-D-25-10954
**Article Type:**	Research Article
**Full Title:**	Social Determinants of Self-Medication with Leftover Antibiotics in Lebanese Households: A Cross-Sectional Study.
**Short Title:**	Social Determinants of Self-Medication with Leftover Antibiotics in Lebanon
**Corresponding Author:**	Reve Rami Khaddaj, BS - MII research<br>Lebanese University Faculty of Pharmacy: Universite Libanaise Faculte de Pharmacie<br>Beirut, LEBANON
**Keywords:**	Social Determinant of Health; Leftover Antibiotics; Self-Medication; Lebanon.
**Abstract:**	Background: Self-medication with leftover antibiotics (LA) is a global health crisis, particularly in contexts of economic and political instability. This study examines the social determinants of health (SDOH) influencing LA use among Lebanese households, focusing on how individuals are born, grow, live, and work.<br>Methods: A cross-sectional online survey of 368 Lebanese adults assessed socio-demographic characteristics, self-medication practices (reasons encouraging and preventing LA use), and SDOH, including the socioeconomic status scale (SES), Discrimination in Medical Setting Scale (DMS), political and economic instability in Lebanon, drug shortage and trusted source of information. Data were analyzed using bivariate tests and multivariable logistic regression.<br>Results: Bivariate analysis revealed significant associations between LA use and male gender (45.0% vs. 33.3%, $p = 0.024$), lower education levels (58.7% vs. 35.1%, $p = 0.002$), chronic disease presence (54.7% vs. 32.2%, $p < 0.001$), older age ($p = 0.028$), and higher household crowding index ($p = 0.018$). Participants affected by political instability ($p < 0.001$), economic crisis ($p < 0.001$), and drug shortages ($p < 0.001$) were more likely to use LAs. In the logistic regression model, chronic disease (OR = 2.711, $p = 0.002$), economic crisis (OR = 2.013, $p < 0.001$), and prior disease experience (OR = 4.085, $p < 0.001$) emerged as significant independent predictors of LA use.<br>Conclusion: These findings show the critical role of socio-economic instability, healthcare access challenges, and experiential factors as key SDOH driving LA practices. The study highlights the urgent need for multi-sectoral interventions addressing economic hardship, improving healthcare access, and enhancing public awareness to mitigate LA misuse and its contribution to antimicrobial resistance in Lebanon.
**Order of Authors:**	Reve Rami Khaddaj, BS - MII research
	Pascale Salameh
	Amal Al-Hajje
	Julia Bou Dib
	Joumana Estephan
	Michele Cherfane
	Reham Kotb
	Diana Nakhoul, BS - MII research
	Reem Awad
	Katia Iskandar
**Additional Information:**	
**Question**	**Response**

Powered by Editorial Manager® and ProduXion Manager® from Aries Systems Corporation

**Financial Disclosure**<br><br>Enter a financial disclosure statement that describes the sources of funding for the work included in this submission. Review the submission guidelines for detailed requirements. View published research articles from *PLOS ONE* for specific examples.<br><br>This statement is required for submission and **will appear in the published article** if the submission is accepted. Please make sure it is accurate.<br><br>**Funded studies**<br>Enter a statement with the following details:<br>• Initials of the authors who received each award<br>• Grant numbers awarded to each author<br>• The full name of each funder<br>• URL of each funder website<br>• Did the sponsors or funders play any role in the study design, data collection and analysis, decision to publish, or preparation of the manuscript?<br><br>Did you receive funding for this work?	The author(s) received no specific funding for this work.
**Competing Interests**<br><br>Use the instructions below to enter a competing interest statement for this submission. On behalf of all authors, disclose any competing interests that could be perceived to bias this work—acknowledging all financial support and any other relevant financial or non-financial competing interests.<br><br>This statement is required for submission and **will appear in the published article** if the submission is accepted. Please make sure it is accurate and that any funding sources listed in your Funding Information later in the submission form are also declared in your Financial Disclosure statement.	The authors have declared that no competing interests exist.

Powered by Editorial Manager® and ProduXion Manager® from Aries Systems Corporation

View published research articles from *PLOS ONE* for specific examples.<br><br>**NO authors have competing interests**<br><br>Enter: *The authors have declared that no competing interests exist.*<br><br>**Authors with competing interests**<br><br>Enter competing interest details beginning with this statement:<br><br>*I have read the journal's policy and the authors of this manuscript have the following competing interests: [insert competing interests here]*<br><br>* typeset	
**Ethics Statement**<br><br>Enter an ethics statement for this submission. This statement is required if the study involved:<br><br>• Human participants<br>• Human specimens or tissue<br>• Vertebrate animals or cephalopods<br>• Vertebrate embryos or tissues<br>• Field research<br><br>Write "N/A" if the submission does not require an ethics statement.<br><br>General guidance is provided below. Consult the submission guidelines for detailed instructions. **Make sure that all information entered here is included in the Methods section of the manuscript.**	- Institutional Review Board at the Lebanese International University under code 2024ERC-020-LIUSOP<br>- Written Informed Consent (at the end of the questionnaire)<br>- Data were analysed anonymously

**Format for specific study types**<br><br>**Human Subject Research (involving human participants and/or tissue)**<br>• Give the name of the institutional review board or ethics committee that approved the study<br>• Include the approval number and/or a statement indicating approval of this research<br>• Indicate the form of consent obtained (written/oral) or the reason that consent was not obtained (e.g. the data were analyzed anonymously)<br><br>**Animal Research (involving vertebrate animals, embryos or tissues)**<br>• Provide the name of the Institutional Animal Care and Use Committee (IACUC) or other relevant ethics board that reviewed the study protocol, and indicate whether they approved this research or granted a formal waiver of ethical approval<br>• Include an approval number if one was obtained<br>• If the study involved *non-human primates*, add *additional details* about animal welfare and steps taken to ameliorate suffering<br>• If anesthesia, euthanasia, or any kind of animal sacrifice is part of the study, include briefly which substances and/or methods were applied<br><br>**Field Research**<br><br>Include the following details if this study involves the collection of plant, animal, or other materials from a natural setting:<br>• Field permit number<br>• Name of the institution or relevant body that granted permission	
**Data Availability**<br><br>Authors are required to make all data underlying the findings described fully available, without restriction, and from the time of publication. PLOS allows rare exceptions to address legal and ethical concerns. See the PLOS Data Policy and FAQ for detailed information.	Yes - all data are fully available without restriction

A Data Availability Statement describing where the data can be found is required at submission. Your answers to this question constitute the Data Availability Statement and **will be published in the article**, if accepted.<br><br>**Important:** Stating 'data available on request from the author' is not sufficient. If your data are only available upon request, select 'No' for the first question and explain your exceptional situation in the text box.<br><br>Do the authors confirm that all data underlying the findings described in their manuscript are fully available without restriction?	
**Describe where the data may be found in full sentences. If you are copying our sample text, replace any instances of XXX with the appropriate details.**<br><br>• If the data are **held or will be held in a public repository**, include URLs, accession numbers or DOIs. If this information will only be available after acceptance, indicate this by ticking the box below. For example: *All XXX files are available from the XXX database (accession number(s) XXX, XXX.).*<br>• If the data are all contained **within the manuscript and/or Supporting Information files**, enter the following: *All relevant data are within the manuscript and its Supporting Information files.*<br>• If neither of these applies but you are able to provide **details of access elsewhere**, with or without limitations, please do so. For example:<br><br>*Data cannot be shared publicly because of [XXX]. Data are available from the XXX Institutional Data Access / Ethics Committee (contact via XXX) for researchers who meet the criteria for access to confidential data.*<br><br>*The data underlying the results presented in the study are available from (include the name of the third party*	The data that support the findings of this study are openly available in Figshare at 10.6084/m9.figshare.28465301

and contact information or URL).<br>• This text is appropriate if the data are owned by a third party and authors do not have permission to share the data.<br><br>* typeset	
Additional data availability information:	Tick here if the URLs/accession numbers/DOIs will be available only after acceptance of the manuscript for publication so that we can ensure their inclusion before publication.

Powered by Editorial Manager® and ProduXion Manager® from Aries Systems Corporation

Manuscript with tables

Click here to access/download;Manuscript;MANUSCRIPT WITH TABLES.docx

Social Determinants of Self-Medication with Leftover Antibiotics in Lebanese Households: A Cross-Sectional Study.

AUTHORS:

Reve Khaddaj[1*], Pascale Salameh[1,2,3], Amal El Hajj[1,2], Julia Bou Dib[4], Joumana Yeretezian[4], Michele Cherfane[5], Reham Kotb[6], Diana Nakhoul[1], Reem Awad[1], Katia Iskandar[2,4,7,8]

AFFILIATIONS:

1. Lebanese University, School of Pharmacy, Beirut, Lebanon. reve-khaddaj@hotmail.com , pascalesalameh1@hotmail.com , amalkeh@hotmail.com , diana.nakhoul@hotmail.com, reem.awad.2010@hotmail.com .
2. INSPECT-LB (Institut National de Santé Publique, d'Épidémiologie Clinique et de Toxicologie-Liban), Beirut, Lebanon. pascalesalameh1@hotmail.com, amalkeh@hotmail.com , katia_iskandar@hotmail.com
3. Department of Primary Care and Population Health, University of Nicosia Medical School, Nicosia, Cyprus; pascalesalameh1@hotmail.com; https://orcid.org/0000-0002-4780-0772
4. Higher Institute of Public Health (ISSP), Saint Joseph University of Beirut, Lebanon; julia.boudib1@usj.edu.lb , joumana.estephan@usj.edu.lb , katia_iskandar@hotmail.com
5. Gilbert and Rose Marie Chagoury School of Medicine, Lebanese American University, Byblos, Lebanon; michellecherfane@gmail.com
6. Environmental and Public Health Department, College of Health Sciences, Abu Dhabi University, Abu Dhabi, UAE. reham.kotb@adu.ac.ae
7. Department of Pharmaceutical Sciences, School of Pharmacy, Lebanese International University, Beirut, Lebanon; katia_iskandar@hotmail.com
8. Faculty of Public Health-Section 2 (CERIPH), Lebanese University, Fanar 90656, Lebanon. katia_iskandar@hotmail.com; https://orcid.org/ 0000-0001-5544-6681

[*] Corresponding author: reve-khaddaj@hotmail.com

ABSTRACT:

Background: Self-medication with leftover antibiotics (LA) is a global health crisis, particularly in contexts of economic and political instability. This study examines the social determinants of health (SDOH) influencing LA use among Lebanese households, focusing on how individuals are born, grow, live, and work.

Methods: A cross-sectional online survey of 368 Lebanese adults assessed socio-demographic characteristics, self-medication practices (reasons encouraging and preventing LA use), and SDOH, including the socioeconomic status scale (SES), Discrimination in Medical Setting Scale (DMS), political and economic instability in Lebanon, drug shortage and trusted source of information. Data were analyzed using bivariate tests and multivariable logistic regression.

Results: Bivariate analysis revealed significant associations between LA use and male gender (45.0% vs. 33.3%, $p = 0.024$), lower education levels (58.7% vs. 35.1%, $p = 0.002$), chronic disease presence (54.7% vs. 32.2%, $p < 0.001$), older age ($p = 0.028$), and higher household crowding index ($p = 0.018$). Participants affected by political instability ($p < 0.001$), economic crisis ($p < 0.001$), and drug shortages ($p < 0.001$) were more likely to use LAs. In the logistic regression model, chronic disease ($OR = 2.711$, $p = 0.002$), economic crisis ($OR = 2.013$, $p < 0.001$), and prior disease experience ($OR = 4.085$, $p < 0.001$) emerged as significant independent predictors of LA use.

Conclusion: These findings show the critical role of socio-economic instability, healthcare access challenges, and experiential factors as key SDOH driving LA practices. The study highlights the urgent need for multi-sectoral interventions addressing economic hardship, improving healthcare access, and enhancing public awareness to mitigate LA misuse and its contribution to antimicrobial resistance in Lebanon.

Keywords: Social Determinant of Health, Leftover Antibiotics, Self-Medication, Lebanon.

1. INTRODUCTION

Antibiotics have revolutionized medical discoveries of the 20th century and shifted the treatment paradigm of bacterial infections [1–4]. While these drugs continue to prevent an estimated 700,000 deaths annually from bacterial infections [5], their effectiveness faces an unprecedented challenge through antimicrobial resistance (AMR). With the rapid global spread of AMR, the World Health Organization (WHO) has classified this ongoing crisis as one of the top three public health threats and leading causes of death worldwide [6]. Recent studies indicated that AMR-related deaths reached approximately 1.27 million in 2019, while nearly 5 million deaths were associated with drug-resistant infections [6]. For decades, the exploitation of these drugs has remained widespread across both developed and developing countries, where they are freely used in human medicine and animal husbandry, serving therapeutic, metaphylactic, and prophylactic purposes in livestock [2,3,7].

A significant driver of AMR is the inappropriate use of antibiotics, predominantly through self-medication (SM) with leftover antibiotics (LA) [8,9]. Defined as unused portions of prescribed antibiotics that patients retain after completing their treatment regimen, LA emerges from over-prescription and incomplete adherence to prescribed courses [10]. The global landscape of antibiotic use shows alarming trends, with consumption surging by over 20% since 2016, particularly in lower- and middle-income countries (LMICs) **(11).** More concerning is that 80% of antibiotics are used outside official healthcare settings, with 20-50% of these uses being deemed inappropriate [12]. The practice of self-medication with LA varies significantly across regions, ranging from 77.5% in Saudi Arabia [13] to 6% in the United Kingdom [14], with intermediate rates of 26.69% in Bangladesh [8] and 44.1% in South America [15].

Beyond its significant role in driving AMR, LA use is a detrimental health practice to individual health and broader public health systems [10,16]. This practice is often associated with multiple health risks, including the use of incorrect or expired medications, increased risk of adverse drug reactions, inappropriate drug selection, incomplete courses of treatment, delayed recovery, and higher risk of complications [17,18].

To better understand the underlying drivers of this public health threat, exploring the underlying associated social determinants of health (SDOH) is a pressing need. [19]. The WHO defines SDOH

as “the non-medical factors that influence health outcomes, including the environments in which individuals live, work, and age, as well as the broader social, economic, and political systems that shape these conditions” [20,21]**.** These determinants include various factors such as education, income level, healthcare accessibility, cultural beliefs, and health literacy. [19–21]. These determinants contribute to health inequities, where health outcomes vary unfairly across socioeconomic groups. Research suggests that SDOH can account for up to 55% of health outcomes, highlighting their importance over healthcare services or personal lifestyle choices. [20,22–24].

Lebanon exemplifies the intersection of economic hardship and health system strain within this global context, presenting a compelling case study of antibiotic misuse. The nation's healthcare system, already burdened by conflict and financial collapse, faces severe challenges, including currency devaluation, medication shortages, and healthcare worker migration [22,23,25]. Recent studies in Lebanon indicate that 23.89% of individuals keep leftover antibiotics at home, with 13.11% expired [10]. The storage, sometimes stockpiling, and subsequent use of LA represents a complex public health challenge stemming from multiple interconnected factors, including healthcare accessibility, prescribing practices, and patient behavior [10,16].

While global research has explored the relationship between SDOH and self-medication with LA [10,26], such as education and knowledge levels of antibiotic use and AMR [27–29], socioeconomic situation [11,24,30,31], access to healthcare services [10,11,32,33], there remains a critical gap in understanding how these factors specifically influence leftover antibiotic use in the Lebanese context. As a middle-income nation grappling with complex healthcare challenges, Lebanon's case highlights the urgent need for targeted research.

This study explores how SDOH shapes self-medication practices with LA in Lebanon, offering critical insights to inform global efforts to combat AMR and support policy development for sustainable healthcare interventions in the region and beyond.

This gap is particularly significant given Lebanon's unique position as a middle-income country experiencing severe economic challenges and complex health system infrastructure [23].

The primary objective of our study is to identify the social determinants that contribute to the use of leftover antibiotics among the Lebanese population.

2. METHODS

Study design

A cross-sectional prospective study was conducted from August 22, 2024, to October 02, 2024, to comprehensively evaluate the SDOH of LA use in Lebanon.

Participants

Eligible participants were Lebanese adults, 18 years of age and above, residing in Lebanon and have internet access.

Questionnaire

The questionnaire consisted of three parts, in addition to the introduction to the survey that encompasses the study topic, objectives, and a statement of consent. The participants were asked if they had ever self-medicated with antibiotic leftovers (Appendix 1).

The first part included the socio-demographic characteristics of participants, such as age, gender, marital status (single and ever married which comprises married, divorced, and widowed), education level (having a university degree or not), health education or not, having children, region of living, distance from their home to the nearest pharmacy (near is considered for a distance lower than 5 Km and far is considered for a distance of 5 Km or more), having or not chronic diseases, area of residence (urban or rural), and the household crowding index, a measure used to assess residential overcrowding, calculated by dividing the number of people living in the house by the total rooms excluding kitchens and bathrooms [34].

The second part focused on examining the practice of self-medication with LA and consisted of two sections:

A. Sources of LA: The participants who self-medicated with LA (answered "yes") were provided four options to indicate the sources of antibiotic leftovers used, including friends, neighbors, family, and household leftovers.
B. Reasons for self-medication or avoidance: The participants who self-medicated with LA (answered "yes") were given 16 predefined answers to identify their motivations to self-medicate. All participants (who answered "yes" or "no" on the question related to LA use), were provided 6 predefined options to capture the reasons that prevent them from self-medication. These questions were inspired by similar studies [35,36].

The third part encompassed the SDOH selected based on their direct relevance to the research question and the socio-political context of Lebanon, with consideration of practical constraints. This part consisted of 5 sections:

1- The Socioeconomic Scale (SES) [37] designed to capture respondent financial strains consists of 8 items. Participants were asked to rate their financial stress over the past month on a scale from 1 to 10, covering areas such as essential expenses and financial stability. Higher scores indicated lower financial stress and better financial well-being. The total possible score ranged from 8 to 80. The scale demonstrated internal consistency (Cronbach's alpha = 0.935).

2- The Discrimination in Medical Setting Scale (DMS) [38] was used to evaluate patients' self-reported experiences of discrimination in healthcare environments. The scale includes 7 items rated on a 5-point Likert scale (1 = Never to 5 = Always), with higher scores reflecting greater perceived discrimination. The total possible score range is 7 to 35. The scale demonstrated strong internal consistency (Cronbach's alpha = 0.89). Participants reported the frequency of discrimination experiences related to their clinical conditions during medical interactions.

3- The socio-economic and political conditions in Lebanon, including factors such as ongoing political conflicts at the time of data collection, political instability, drug shortages, and the ongoing economic crisis. The assessment aimed to capture the contextual challenges influencing healthcare behaviors during periods of national crisis. Each item was rated on a **5-point scale** (1 = No influence, 5 = very strong influence), where **higher scores indicate a greater perceived impact** of socioeconomic and political factors on their SM behavior.

4- Trusted source of information consisted of 9 options adapted from previous studies [39–42]. Respondents were asked to indicate whether they trusted each source, using a **yes/no** format. It was formulated to assess the source of information that participants relied on when using LA without a doctor's prescription.

5- Level of knowledge of the participants about antibiotic use, antibiotic resistance, and LA use risks: it was divided into three sub-sections, each using a standardized 5-point Likert scale ("strongly disagree" to "strongly agree"). For all sub-sections, the scoring methodology followed these principles. Respondents rated their agreement on a 5-point Likert scale ranging from "strongly disagree" to "strongly agree." Correct responses were assigned one point based on the statement type. For true statements, selecting "agree" or "strongly agree" was correct, while for false statements, selecting "disagree" or "strongly disagree" earned a point. Neutral responses

were considered incorrect. Reverse coding was applied where necessary for negatively phrased statements. Participants scoring 70% or higher were classified as having good knowledge [43]. The sub-sections were structured as follows:

- Knowledge of antibiotic use sub-section included 18 statements adapted from previous studies [28,44–48]. It focuses on antibiotic effects, use, and side effects.
- Knowledge of antibiotic resistance sub-section comprised 9 statements adapted from previous research [28,44–46]. It focuses on AMR spread mechanisms, global impact, personal health implications, transmission patterns between humans and animals, and contributing factors to resistance development.
- Knowledge of risks associated with LA use sub-section included 12 statements adapted from previous studies [36,49]. It assesses the knowledge about risks and potential dangers associated with SM using leftover antibiotics, including understanding inappropriate drug use, safety concerns, diagnostic errors, adverse effects, therapeutic failures, and economic consequences.

Data collection

Data were collected using an online questionnaire formulated in English and created on Google Forms, a cloud-based survey powered by Google™. A forward translation was first performed from English into Arabic and then back-translated into English. The two English versions were compared, with minor discrepancies corrected by consensus between the translators and the authors. A pilot test of the questionnaire included 15 participants from different age groups, genders, socio-economic and educational backgrounds to identify any potential ambiguities in language or expressions. Subsequent feedback prompted necessary revisions for clarity and cultural relevance. The link to the questionnaire was shared using various social media platforms, including WhatsApp, Facebook, Instagram, and LinkedIn. Data were collected using the snowball sampling technique [50].

Ethical approval

This study, approved by the Institutional Review Board at the Lebanese International University under code 2024ERC-020-LIUSOP, adhered to the Declaration of Helsinki [51]. Before filling out the online survey, participants were briefed about the study objectives and their right to withdraw at any time. Informed consent was obtained from all participants, by including a consent statement

at the beginning of the survey. Participants were required to read the statement and click a checkbox indicating their agreement to participate to enable proceeding with the survey. Participants did not receive any financial reward for their participation. The online survey was anonymous and voluntary. Collected data were encrypted, stored in password-protected computers, and presented as de-identified electronic files in Microsoft Excel and SPSS.

Sample size calculation

The minimal sample size was calculated using Epi-Info version 7.2.6. Based on a previously published study in Lebanon [10], the prevalence of LA use among the Lebanese population was 23.89%. The minimum necessary sample was n=279 participants considering an alpha error of 5% and a power of 95%. A minimum sample of 300 participants was targeted to account for potential missing values.

Statistical analysis

Data were analyzed using SPSS version 26. Descriptive statistics were performed to summarize the sociodemographic characteristics, the impact of the Lebanese crisis on the decision to use leftover antibiotics, the SDOH, and knowledge regarding antibiotics, antibiotic resistance, and risk of LA use. Continuous variables were summarized using means standard deviations (SD), and medians, while categorical variables were presented as percentages.

Bivariate analysis was conducted to examine the association between leftover antibiotic use (dependent variable) and other independent variables. For categorical variables, Chi-square tests were used, and for continuous variables, the student's t-test was applied. A p-value < 0.05 was considered statistically significant.

Logistic regression was employed with the use of leftover antibiotics as the dependent variable. Independent variables with a p-value < 0.2 from the bivariate analysis were included in the model using the Enter method. The Omnibus test was performed to assess model significance, while the correlation matrix was examined to check for collinearity. The Hosmer and Lemeshow tests were used to evaluate the model's goodness-of-fit, and Nagelkerke R^2 was reported to determine the explanatory power of the model.

3. RESULTS

Socio-demographic characteristics

Table 1 summarizes the socio-demographic characteristics of participants (N=368) and their association with self-medication using LA. A significant association was observed with gender ($p = 0.024$), as males were more likely to self-medicate (45.0%) than females (33.3%). Education level also showed significance ($p = 0.002$), with participants holding a university degree reporting the lowest self-medication rate (35.1%). The presence of chronic disease was strongly associated with self-medication behavior ($p < 0.001$). Although marital status and parenthood showed no significant associations, ever-married participants (40.6%) and those with children (40.7%) exhibited higher self-medication rates. No significant relationship was found with the field of study, region category, area of living, or proximity to a pharmacy. However, participants living near a pharmacy reported higher self-medication (39.2%) than those farther away (24.1%, $p = 0.108$). Age (45.50 ± 13.599 years in non-LA users, and 48.72 ± 13.618 years in LA users, $p=0.028$) and household crowding index (HCI: 0.904 ± 0.471 in non-LA users, and 1.061 ± 0.666 in LA users, $p = 0.018$) were significantly associated with LA use.

Table 1 : Socio-Demographic Characteristics of Participants (N=368)

Self-medication with Leftover antibiotics (N= 368)			
**Variables**	**No**<br>**n=228**	**Yes**<br>**n=140**	**p-value**
**Gender**			
Male	82 (55.0)	67 (45.0)	0.024*
Female	146 (66.7)	73 (33.3)	
**Marital Status**			
Single	63 (70.0)	27 (30.0)	0.071
Ever married	165 (59.4)	113 (40.6)	
**Have Children**			
No	81 (67.5)	39 (32.5)	0.128
Yes	147 (59.3)	101 (40.7)	
**University Degree**			
No	19 (41.3%)	27 (58.7%)	0.002*
Yes	209 (64.9%)	113 (35.1%)	
**Field of Study**			
Health	46 (66.7)	23 (33.3)	0.371
Non-health	182 (60.9)	117 (39.1)	
**Suffering From Chronic Disease**			
No	185 (67.8)	88 (32.2)	<0.001**
Yes	43 (45.3)	52 (54.7)	
**Region Category**			
Beirut	38 (61.3)	24 (38.7)	
North	39 (69.6)	17 (30.4)	
Mount Lebanon	103 (61.7)	64 (38.3)	0.474
Beqaa	27 (64.3)	15 (35.7)	
South	21 (51.2)	20 (48.8)	
**Area of Living**			
Urban	151 (62.1)	92 (37.9)	0.920
Rural	77 (61.6)	48 (38.4)	
**Distance From Home to Nearest Pharmacy**			
Near	206 (60.8)	133 (39.2)	0.108
Far	22 (75.9)	7 (24.1)	
	**Mean (SD)**		
**Age (years)**	45.500 ± 13.599	48.720 ± 13.618	0.028*
**House Crowding Index (HCI)**	0.907 ± 0.471	1.061 ± 0.666	0.0[illegible]

* Indicates significant value ($p < 0.05$); ** Indicates highly significant value on a level 1% ($p \leq 0.001$);
Near: < 5 km; Far ≥ 5km; SD: Standard Deviation; Ever Married: Married, widowed, or divorced.

Practice of self-medication

Figure 1 shows that the primary source of LA used is household antibiotics (76.3%) followed by those borrowed from family (32.4%). Relying on neighbors and friends as a source of LA was the least (7.9% and 13.7% respectively).

Figure 2 indicates that participants cited various reasons for engaging in this practice. The most frequent reasons include having a known disease (87.9%) and experiencing minor ailments

(89.2%), followed by the availability of leftover antibiotics (70.7%) and convenience (69.3%). Knowledge about medications (85.7%) and inadequate time to visit a doctor (73.6%) were also predominant factors. Additionally, quick relief (83.6%) and saving time (58.6%) were notable motivations, while privacy (26.4%) and distrust in doctors (12.9%) were reported by fewer participants.

Figure 3 compares the reasons that prevent participants from self-medicating with LA between those who self-medicate with LA and those who do not. Among non-users of LA, the most common reasons include the belief that there is no need to self-medicate (70.6%), concerns about the risk of side effects (64.9%), and a preference for medical consultation (63.8%). Additionally, 63.4% of non-users trust doctors for a better diagnosis, while 58% cited a lack of knowledge as a reason to avoid SM. In contrast, these concerns were less common among those who self-medicate, with only 29.4% seeing no need for self-medication, 35.1% worried about side effects, and 36.2% preferring medical consultation. Similarly, fewer self-medication users trusted a doctor’s diagnosis (36.6%) or were aware of their lack of knowledge (42.3%). Other safety concerns were also more frequently reported by non-LA users (60%) compared to LA users (40%).

Social determinant of health

Table 2 indicated a significant difference observed in DMS scores, with those who used LAs scoring lower than non-users ($p = 0.029$). Participants' perceptions of the **political situation, drug shortage,** and **economic crisis** in Lebanon were significantly associated with LA use ($p < 0.001$). Additionally, the ongoing political conflict in Lebanon was significantly associated with LA use ($p = 0.047$). Moreover, those who used LAs had significantly lower **knowledge of antibiotics** compared with non-users ($p = 0.005$). No significant associations were found between LA use and the **SES scale** ($p = 0.130$), **knowledge about AMR** ($p = 0.734$), and **knowledge about the risks of LA use** ($p = 0.200$). Participants scoring 70% or higher were classified as having good knowledge [43] which means that a score considered good knowledge is 6 for AMR knowledge, 12 for antibiotics knowledge, and 8 for risk of LA use knowledge.

Table 2 : Social Determinants of Leftover Antibiotic Use

Leftover Antibiotic Use			
**Variables**	**No**<br>**n=228**	**Yes**<br>**n=140**	**p-value**
**Mean (SD)**			
**SES Scale**	38.065 ± 17.809	35.250 ± 16.366	0.130
**DMS Scale**	31.017 ± 4.225	29.892 ± 5.061	0.029*
**Lebanese socio-economic and political context**			
Political Situation	1.910 ± 1.251	2.540 ± 1.547	<0.001**
Drug Shortage	2.270 ± 1.428	3.250 ± 1.541	<0.001**
Economic Crisis	2.010 ± 1.282	3.050 ± 1.485	<0.001**
Ongoing Political Conflict	1.790 ± 1.265	2.080 ± 1.368	0.047*
**Level of knowledge**			
AMR	4.350 ± 2.831	4.450 ± 2.844	0.734
Antibiotics	11.050 ± 4.618	9.700 ± 4.039	0.005*
The risks of LA use	9.12 ± 2.93	8.68 ± 3.05	0.200

*Indicates significant value ($p < 0.05$); **Indicates highly significant value on a level 1% ($p \leq 0.001$)
SD: Standard Deviation; SES: Socioeconomic Status; DMS: Discrimination in Medical Setting;
AMR: Anti-microbial Resistance; LA: Leftover Antibiotics

Table 3 showed a significant association between LA use and participants' **previous experience of the disease** ($p < 0.001$), with individuals who had prior disease experience being more likely to use leftover antibiotics (49.8%) than those without such experience (19.6%). Additionally, reliance on **a family member's opinion** was significantly associated with LA use ($p < 0.001$), as 51.7% of those influenced by family opinions reported LA use compared to 31.6% who did not. Other factors, such as the role of **pharmacists, academic knowledge, internet usage, reading materials, social media, mass media,** and **artificial intelligence**, showed no statistically significant association with LA use ($p > 0.05$).

Table 3: Trusted Source of Information About Leftover Antibiotic Use

Leftover Antibiotic Use	No<br>n=228	Yes<br>n=140	
**Variables**	**Frequency (%)**		**p-value**
**Pharmacist**			
No	12 (50.0)	12 (50.0)	0.212
Yes	216 (62.8)	128 (37.2)	
**Previous Experience with The Disease**			
No	115 (80.4)	28 (19.6)	<0.001**
Yes	113 (50.2)	112 (49.8)	
**Academic Knowledge**			
No	99 (59.3)	68 (40.7)	0.335
Yes	129 (64.2)	72 (35.8)	
**A Family Member's Opinion**			
No	171 (68.4)	79 (31.6)	<0.001**
Yes	57 (48.3)	61 (51.7)	
**Internet**			
No	117 (66.9)	58 (33.1)	0.065
Yes	111 (57.5)	82 (42.5)	
**Reading Materials**			
No	168 (64.9)	91 (35.1)	0.076
Yes	60 (55.0)	49 (45.0)	
**Social Media**			
No	198 (62.9)	117 (37.1)	0.386
Yes	30 (56.6)	23 (43.4)	
**Mass Media**			
No	204 (62.2)	124 (37.8)	0.787
Yes	24 (60.0)	16 (40.0)	
**Artificial Intelligence**			
No	216 (62.4)	130 (37.6)	0.460
Yes	12 (54.5)	10 (45.5)	

**Indicates highly significant value on a level 1% ($p \leq 0.001$)

Multivariable analysis

Table 4 showed the results of the logistic regression which revealed several significant predictors of the outcome (the use of LA). Relying on previous disease experience as a source of information emerged as the strongest predictor ($\beta = 1.407$, 95% CI [2.242;7.442], $p < 0.001$), with an odds ratio indicating that individuals with prior experience with the disease were over four times more likely to experience the outcome. The presence of a chronic disease significantly increased the likelihood of the outcome ($\beta = 0.997$, 95% CI [1.443;5.094], $p = 0.002$), while the economic crisis also demonstrated a substantial effect ($\beta = 0.700$, 95% CI [1.383;2.931], $p < 0.001$). Drug shortage showed marginally significant associations ($\beta = 0.219$, 95% CI [0.980-1.582], $p = 0.073$). Notably, demographic factors including gender, marital status, having children, age, crowding index, and education level did not significantly predict the outcome. Similarly, the discrimination scale, SES

scale, knowledge about antibiotic use and risks of LA use, family opinion, pharmacist, Internet, and reading materials showed no significant associations with the outcome. Moreover, the political conflicts in Lebanon and the region and the difficult political situation in Lebanon did not show a significant correlation with LA use.

Table 4: Logistic Regression Using Self-Medication with LA as The Dependent Variable

Variable	B	Odds ratio	p-value	95% CI
Gender (male*/female)	-0.261	0.770	0.352	0.445-1.334
Marital Status (single*/ever married)	-0.185	0.831	0.728	0.293-2.356
Having Children (No*/yes)	0.997	1.448	0.435	0.571-3.673
Age	-0.007	0.993	0.593	0.969-1.018
Household Crowding Index	0.350	1.419	0.158	0.872-2.309
University Degree (No*/yes)	-0.537	0.585	0.208	0.254-1.347
Chronic Diseases (No*/yes)	0.997	2.711	0.002**	1.443-5.094
SES Scale	0.005	1.005	0.564	0.989-1.021
Discrimination Scale	-0.020	0.980	0.535	0.921-1.044
Political Situation	-0.321	0.726	0.132	0.478-1.101
Economic Crisis	0.700	2.013	<0.001***	1.383-2.931
Drugs Shortage	0.219	1.245	0.073†	0.980-1.582
Ongoing Political Conflict	-0.233	0.792	0.137	0.582-1.077
Knowledge About Antibiotic Use	-0.036	0.965	0.252	0.908-1.026
Knowledge About Risks Of LA Use	-0.040	0.960	0.354	0.881-1.046
Pharmacist (No*/yes)	-0.553	0.575	0.285	0.209-1.584
Previous Experience with The Disease (No*/yes)	1.407	4.085	<0.001***	2.242-7.442
Family Opinion (No*/yes)	0.155	1.167	0.595	0.660-2.065
Internet (No*/yes)	0.121	1.129	0.676	0.639-1.994
Reading Material (No*/yes)	0.168	1.183	0.596	0.635-2.206

*Reference variable; CI: Confidence Interval
**Indicates significant value ($p < 0.05$); ***Indicates highly significant value on a level 1% ($p \leq 0.001$);
†Indicated marginally significant value ($p < 0.10$)
LA: Leftover Antibiotics   SES: Socioeconomic Status

4. DISCUSSION:

This study examined the social determinants of LA use in Lebanon, revealing complex interactions among health-system, economic, and social factors. The SDOH selection was guided by the WHO's conceptual framework [20], which provides a structured approach to understanding how various social factors influence health outcomes through both structural and intermediary determinants. The selected SDOH included traditional socioeconomic indicators (education, household crowding, economic status) and context-specific elements reflective of Lebanon's ongoing crisis (political instability, drug shortages, healthcare discrimination). The inclusion of discrimination and healthcare access measures was especially pertinent given Lebanon's complex sectarian landscape [52] and their documented impact on healthcare delivery and outcomes [53–55]**.** Incorporating family influence and information sources intended to capture the role of social networks in Lebanese healthcare decision-making, an aspect often underexplored in traditional SDOH frameworks but particularly relevant in Middle Eastern contexts. This integrated approach enabled a comprehensive examination of how multiple layers of social determinants influence medication behaviors in a fragmented healthcare system [23].

The study showed a concerning 38% prevalence of LA use, significantly exceeding the rates 23.89% rate reported in a recent Lebanese study [10]. These results position Lebanon as an intermediate between nations with robust regulatory frameworks, like the United Kingdom (6%) [14] and Saudi Arabia (77.5%) [13]. The reliance on leftover antibiotics represents a coping mechanism, as documented in two Lebanese studies [10,56], where individuals navigate the dual pressures of financial constraints and inadequate healthcare access [57]**.** This adaptation to economic constraints presents a particular challenge for antimicrobial stewardship efforts, as individuals may prioritize immediate medication access over long-term resistance concerns [58].

Several critical insights into reasons for self-medication practices in Lebanon, with findings showing 73.6% self-reported medication knowledge compared with 48.8% in Iran [59], 42.3% in Serbia [60], and 23.59% in Nepal [42]. Accessibility factors played a predominant role, with 69.3% citing convenience and 70.7% antibiotic availability as motivators. A total of 83.6% of participants indicated immediate relief-seeking behavior, a high rate exceeding those reported in other countries, with 28% in Saudi Arabia [13] and 19.1% in Nepal [42], while time-saving

considerations motivated 58.6% of respondents. Additionally, feedback from participants about reasons for avoiding LA use highlighted higher rates of concerns compared with LA users, such as preferring medical consultations, trusting physicians for better diagnoses, and fear of side effects. Safety concerns were noted by 40% of LA users and 60% of non-users, while 70.6% of non-users cited no need for self-medication compared to 29.4% of LA users. These patterns reflect the complex interplay between insufficient regulatory oversight, compromised healthcare access, and economic constraints in shaping medication behaviors. The prevalence is particularly alarming given the spread of AMR in Lebanon [61] and its potential health and environmental impact in an AMR-endemic Middle-Eastern Mediterranean region [62].

The predominant sources of LA reveal concerning patterns, with household antibiotics (76.3%) and family borrowing (32.4%) being the main sources. This high rate of household antibiotic storage suggests potential issues with prescription quantities, treatment adherence, or deliberate stockpiling behaviors. The family borrowing pattern reinforces the strong role of social networks in medication access, creating informal channels that bypass professional healthcare guidance. The influence of social networks, particularly family opinions, reflects the cultural context in which health decisions occur. Moreover, the influence of family opinions emerged as a significant factor considered to impact SM with LA (51.7% of LA users rely on a family member's opinion, $p<0.001$). This shows that family trust plays a huge role in health decisions: in cultures with strong family bonds, people often refer to their families for healthcare advice. This amplifies the need to educate the general population to prevent antibiotic misuse [63,64].

While this influence was not statistically significant in multivariate analysis, the qualitative patterns suggest that health decision-making in Lebanon occurs within a complex social framework that can either reinforce or challenge formal medical advice. This understanding is crucial for developing culturally appropriate interventions that acknowledge the role of family networks in medication practices, particularly in contexts where traditional healthcare systems may be compromised by economic and political instability. The reliance on family for health-related guidance as a trusted source of information may be particularly pronounced in cultures with strong familial ties, emphasizing the need for targeted educational interventions within these networks to mitigate inappropriate antibiotic use [63,64]. Conversely, other sources such as trust in pharmacists, academic knowledge, internet use, and social media showed no significant

associations with LA use. This finding raises important questions about the effectiveness of these channels in shaping antibiotic consumption behaviors. For instance, despite the potential for pharmacists to provide valuable guidance on antibiotic use, their influence appears limited in this context. This could be attributed to barriers such as accessibility issues or a lack of proactive communication from healthcare professionals regarding proper antibiotic usage and disposal practices [65]. The absence of significant correlations with digital information sources also highlights a potential gap in public health messaging. As many individuals increasingly turn to online platforms for health information, it is crucial to assess the quality and reliability of such resources. The lack of engagement through these channels may suggest that public health campaigns need to adapt their strategies to better reach and educate populations about the risks associated with leftover antibiotics [36,66]**.**

Personal health experiences emerged as the strongest predictors, particularly considering previous disease experience as a trusted source of information and chronic disease status. The results suggest that individuals with prior disease experiences develop increased self-efficacy in managing similar symptoms, potentially reducing their perceived need for professional healthcare advice [67]. Studies from Nepal [42] and India [64] reported similar patterns, suggesting that this may be a broader phenomenon in healthcare systems with limited regulatory oversight. This normalization of antibiotic self-management is particularly concerning in the context of AMR, as it may accelerate the development of resistant bacterial strains through inappropriate use patterns and create reservoirs of resistant organisms within communities [7].

Gender emerged as a significant factor, with males showing higher LA use (45%) compared to females (33.3%). This gender disparity warrants further investigation, potentially reflecting differences in healthcare-seeking behaviors, risk perception, or socioeconomic factors affecting healthcare access between men and women in the Lebanese context. These findings oppose to previous studies in Lebanon [10,55] where gender-based differences in medication behaviors were observed. Similar studies conducted in Nepal [42] and Serbia [60] found no gender influence on self-medication practice. Additionally, the significant association of the household crowding index with LA use highlights the role of living conditions in medication behaviors. This finding suggests that household dynamics and living arrangements may influence medication sharing and storage practices, adding another layer to our understanding of social determinants.

Socio-economic and political stressors, particularly the ongoing economic crisis and widespread drug shortages, emerged as strong predictors influencing LA utilization. Interestingly, the SES scale which typically includes indicators of the individual financial strains, was not significantly associated with LA. While socioeconomic factors are often strong predictors of healthcare behaviors [68,69] as they reflect an individual's ability to access to resources, our findings suggest a more complex dynamic in the Lebanese context. The severe devaluation of the Lebanese pound and its impact on purchasing power has made proper healthcare access increasingly challenging for many residents, pushing them toward potentially dangerous self-medication practices [30]. While numerous studies conducted in Lebanon [10], Saudi Arabia [39] and others [63,70] have documented the influence of the individual socio-economic status on health behavior including antibiotic misuse, our study reveals a different pattern. The lack of significance in SES correlation may stem from the equalizing effects of the Lebanon's ongoing economic crisis and drug shortages, which impact individuals across all socio-economic strata [30]. This crisis-specific context might overshadow traditional SES distinctions, as immediate situational factors such as drug shortage can take precedence over long-term socio-economic markers. Moreover, the SES scale may not fully capture critical variables relevant to LA use, such as trust in the healthcare system or informal medication sources, which may vary independently of SES. These findings suggest that LA use trends are shaped by a complex interplay of structural vulnerabilities and situational challenges, necessitating targeted interventions that address specific barriers like drug shortages and healthcare access limitations, rather than broader SES categorizations. The patterns reflect systemic challenges, including limited public health infrastructure, inadequate pharmaceutical supply chain regulation, and insufficient safety nets for economically disadvantaged populations.

The situation is further complicated by Lebanon's fragmented healthcare surveillance system and loose regulatory oversight of antibiotic dispensing, creating an environment conducive to inappropriate antibiotic use [23]. Addressing these issues requires a systematic approach that includes strengthening healthcare systems, improving the affordability and availability of medications, and implementing targeted public health interventions to reduce reliance on inappropriate self-medication practices.

Earlier national studies indicated that lower educational attainment was linked to higher rates of self-medication and antibiotic misuse [10,55]. Our findings align with previous research in this area showing that participants with university degrees are less likely to self-medicate with LA (35.1% of university graduates use LA compared to 58.7% without university degrees, p=0.008). Research from Saudi highlighted that inadequate knowledge about antibiotic resistance was prevalent among lower-educated individuals, leading to increased self-medication practices, mirroring our results where LA users had significantly lower knowledge about antibiotics [49]. Similarly, a cross-sectional study in Italy demonstrated that individuals with lower educational levels were more likely to engage in self-medication and exhibited poorer knowledge regarding antibiotic use, reinforcing the global trend observed in our research [18].

The significant association with education level presents a complex picture, particularly when considered alongside age-related patterns. The relationship between these demographic factors and LA use suggests the need for targeted educational interventions that consider both age-specific behaviors and educational background.

Our analysis revealed nuanced relationships between antibiotic knowledge and LA use. While knowledge of antibiotic resistance and LA use risks did not correlate significantly with LA use, there was a clear association between general knowledge about antibiotics and their usage patterns. Participants who reported using LA exhibited significantly lower knowledge scores compared with non-users. These findings suggest that enhanced knowledge about antibiotics correlates with more responsible usage behaviors, reducing the likelihood of retaining unused medications.

This pattern aligns with existing literature on education's role in antibiotic use. A Lebanese study found that inadequate understanding of antibiotic usage directly contributed to self-medication practices among various demographic groups [10]. Research from Cyprus reinforced this, showing how improved awareness and attitudes toward antibiotics could significantly reduce self-medication behaviors [45]. International studies have consistently shown similar patterns - from Serbia, where medical students' self-medication practices varied with their understanding of antibiotic protocols [60], to Saudi Arabia, where widespread misconceptions contributed to inappropriate use [28], and Ethiopia, where poor knowledge correlated with medication misuse [46]. A multicentric survey across 14 European countries further supported these findings,

revealing that 84% of the public lacked knowledge regarding appropriate antibiotic use, with only 37% receiving antibiotic-related information in the previous year [71].

Another significant finding was the inverse relationship between perceived discrimination and LA use, contrasting with international literature. While other studies have frequently identified higher perceived discrimination as a barrier to healthcare access, often resulting in increased self-medication practices [53,56,65], our findings in Lebanon's healthcare context suggest that individuals who perceive less discrimination have better access to formal healthcare services. This access could facilitate the accumulation of leftover antibiotics through formal prescriptions, potentially due to issues with prescription practices, medication over-dispensing, or lack of patient adherence to prescribed regimens. The relationship between discrimination and LA use reveals a unique dynamic within Lebanon's sectarian healthcare system. LA users reported lower scores on the discrimination scale, suggesting that reduced discrimination correlates with greater access to medications. This aligns with the hypothesis that Lebanon's healthcare system, which often stratifies access based on socio-political affiliations and network ties, can unintentionally exacerbate inappropriate antibiotic usage by enabling those with better access to accumulate antibiotics. This paradoxical relationship underscores the complexity of healthcare access in Lebanon, where systemic and contextual factors may overshadow traditionally observed dynamics of healthcare discrimination. These findings warrant further investigation into prescription and dispensing practices within Lebanon's healthcare system to better understand their implications for antibiotic stewardship. Addressing these structural issues, including over-prescription and inadequate monitoring of antibiotic use, is critical for minimizing antibiotic resistance risk and promoting more rational medication use.

Limitations:

Several methodological limitations warrant consideration when interpreting this study's findings. First, the cross-sectional design inherently precludes causal inference regarding the relationships between SDOH and LA use. Second, the snowball sampling technique used, while necessary given the challenging research context, may have introduced selection bias by potentially overrepresenting certain social networks and demographic groups. Although efforts were made to diversify recruitment across regions and socioeconomic strata, the age groups, socioeconomic

groups, and areas of living were not equally represented. For this reason, the generalizability of our findings to the broader Lebanese population should be approached with caution. Third, the reliance on self-reported data presents another significant limitation, potentially introducing social desirability and recall biases. While the questionnaire was designed to minimize leading questions and ensure anonymity, participants' responses may have been influenced by their perceptions of socially acceptable behavior regarding antibiotic use. Fourth, the timing of data collection during a period of war, significant political conflicts, and economic crisis in Lebanon may have affected both response patterns and the representativeness of typical healthcare-seeking behaviors. Fifth, while our selection of social determinants was theoretically grounded in established SDOH frameworks, other unmeasured factors may influence LA use in the Lebanese context. Sixth, **residual confounding bias remains a potential limitation when examining the association between social determinants and LA use. Factors such as socioeconomic status, education, and healthcare accessibility may not always be fully captured or accurately measured, leading to an incomplete adjustment in statistical models. This can distort the observed relationship, either overestimating or underestimating the true effect of social determinants on LA use. For instance, if income level is not adequately accounted for, its residual influence may create a misleading association between education and LA use. Addressing this bias requires robust data collection and advanced analytical techniques to ensure a more accurate interpretation of findings. This limitation was addressed by doing a logistic regression.**

This study was one of the first in Lebanon that examined the SDOH of LA use. It provided crucial insights regarding the impact of SDOH on the LA use filling an important gap in the literature. Additionally, the large sample size studied was an important factor. Moreover, the completed data obtained (no missing data) is also an important strength of this study. Longitudinal studies are urgently needed to examine the temporal dynamics between social determinants and antibiotic use behaviors, particularly in the context of Lebanon's rapidly evolving socioeconomic landscape. Such studies would be invaluable in establishing causal relationships and understanding how changes in social conditions affect medication practices over time.

Future directions

Future research should prioritize assessing the impact of regulatory interventions on antibiotic dispensing, particularly during economic crises that limit healthcare access. Mixed-methods approaches can provide both quantitative data and qualitative insights into implementation challenges. Investigating healthcare provider prescribing behaviors and their link to LA availability is essential, especially in resource-constrained settings where social and economic pressures shape decision-making. Comparative studies across diverse cultural contexts can clarify the relationship between social determinants and healthcare systems, guiding culturally tailored interventions. Implementation research should evaluate the effectiveness of strategies targeting social determinants, including family-centered approaches that acknowledge the role of social networks in medication decisions. Additionally, strengthening pharmaceutical regulations is crucial, ensuring they do not create further barriers for vulnerable populations. Surveillance of antibiotic consumption and resistance patterns must be reinforced, given Lebanon's role in regional AMR spread. Healthcare providers need support in managing patient expectations and prescribing practices, particularly during economic hardships. Furthermore, a coordinated regional approach is necessary to address AMR, as Lebanon's experience highlights how social determinants contribute to antibiotic misuse, influencing broader regional resistance patterns.

5. CONCLUSION:

This study reveals complex social determinants influencing LA use in Lebanon, with a concerning 38% prevalence rate. Key determinants include previous disease experience, chronic conditions, economic crisis impacts, and healthcare system factors. The findings highlight paradoxical relationships between discrimination, education, and LA use that differ from international patterns. While knowledge about antibiotics shows some protective effects, structural factors like economic crises and drug shortages appear to override traditional socioeconomic determinants. In Lebanon's challenging context, LA use emerges as a coping mechanism shaped by multiple intersecting social factors rather than individual characteristics alone. These findings emphasize the urgent need for a comprehensive approach to addressing LA use that considers individual behaviors and systemic challenges, particularly in resource-constrained settings experiencing economic and political instability. Strengthening pharmaceutical regulations, improving surveillance systems, and supporting healthcare providers must be balanced with ensuring healthcare access for vulnerable populations. The complex interplay of social determinants identified in this study provides crucial

insights for developing culturally appropriate, context-specific interventions to promote more rational antibiotic use while acknowledging the unique challenges the Lebanese healthcare system faces.

LIST OF ABBREVIATIONS:

AMR: Antimicrobial Resistance

CI: Confidence Interval

DMS: Discrimination in Medical Setting

LA: Leftover Antibiotic

LMIC: Lower- and Middle-Income Countries

SD: Standard Deviation

SES: Socioeconomic Status

SDOH: Social Determinant of Health

SM: Self-Medication

WHO: World Health Organization

DECLARATIONS:

Ethical Approval: This study, approved by the Institutional Review Board at the Lebanese International University under code 2024ERC-020-LIUSOP, adhered to the Declaration of Helsinki [51].

Data Availability: The data that support the findings of this study are openly available in Figshare at 10.6084/m9.figshare.28465301 [72].

Acknowledgment: Not applicable

Author Contributions:

RK and KI: study conception and design. RK acquisition of data. RK, KI, JBD, and JY: analysis and interpretation of results. RK, KI, JBD, AND JY: Software, JBD, and KI: visualization. RK and KI: investigation. KI project administration. RK and KI: Writing an original manuscript draft. AH, PS MC, RK, DN, RA: revision and editing it critically for important intellectual content. KI,

RK, PS, AH, MC, RK, DN, RA: validation. KI: supervision. All authors contributed to the article and approved the submitted version.

Conflict of Interest:

The authors declare that the research was conducted in the absence of any commercial or financial relationships that could be construed as a potential conflict of interest.

Funding:

No funding was received for the completion of this research. The study was conducted without financial support from any funding agencies or organizations.

REFERENCES:

1. Adedeji WA. THE TREASURE CALLED ANTIBIOTICS. Ann Ib Postgrad Med. 2016 Dec;14(2):56–7.

2. Salam MA, Al-Amin MY, Salam MT, Pawar JS, Akhter N, Rabaan AA, et al. Antimicrobial Resistance: A Growing Serious Threat for Global Public Health. Healthcare (Basel). 2023 Jul 5;11(13):1946.

3. Williams-Nguyen J, Sallach JB, Bartelt-Hunt S, Boxall AB, Durso LM, McLain JE, et al. Antibiotics and Antibiotic Resistance in Agroecosystems: State of the Science. J Environ Qual. 2016 Mar;45(2):394–406.

4. Iskandar K, Murugaiyan J, Hammoudi Halat D, Hage SE, Chibabhai V, Adukkadukkam S, et al. Antibiotic Discovery and Resistance: The Chase and the Race. Antibiotics (Basel). 2022 Jan 30;11(2):182.

5. Klein EY, Impalli I, Poleon S, Denoel P, Cipriano M, Van Boeckel TP, et al. Global trends in antibiotic consumption during 2016-2023 and future projections through 2030. Proc Natl Acad Sci U S A. 2024 Dec 3;121(49):e2411919121.

6. Antimicrobial Resistance Collaborators. Global burden of bacterial antimicrobial resistance in 2019: a systematic analysis. Lancet. 2022 Feb 12;399(10325):629–55.

7. Iskandar K, Molinier L, Hallit S, Sartelli M, Catena F, Coccolini F, et al. Drivers of Antibiotic Resistance Transmission in Low- and Middle-Income Countries from a ‘One Health’ Perspective-A Review. Antibiotics (Basel). 2020 Jul 1;9(7):372.

8. Biswas M, Roy MN, Manik MIN, Hossain MS, Tapu STA, Moniruzzaman M, et al. Self-medicated antibiotics in Bangladesh: a cross-sectional health survey conducted in the Rajshahi City. BMC Public Health. 2014 Dec;14(1):847.

9. Antimicrobial Resistance [Internet]. World Health Organization (WHO); 2023. Available from: https://www.who.int/news-room/fact-sheets/detail/antimicrobial-resistance

10. Saadeh W, Chaccour S, Rahme D, Lahoud N, Saleh N. The hidden dangers lurking at home: Unveiling the prevalence of leftover antibiotics and its associated factors among Lebanese households. Public Health Pract (Oxf). 2024 Jun;7:100485.

11. Mendoza AMB, Maliñana SAA, Maravillas SID, Moniva KC, Jazul JP. Relationship of self-medication and antimicrobial resistance (AMR) in low- and middle-income countries (LMICs): a scoping review. J Public Health Emerg. 2025 Mar;9:6–6.

12. Browne AJ, Chipeta MG, Haines-Woodhouse G, Kumaran EPA, Hamadani BHK, Zaraa S, et al. Global antibiotic consumption and usage in humans, 2000–18: a spatial modeling study. The Lancet Planetary Health. 2021 Dec;5(12):e893–904.

13. Eltom EH, Alanazi AL, Alenezi JF, Alruwaili GM, Alanazi AM, Hamayun R. Self-medication with antibiotics and awareness of antibiotic resistance among the population in Arar city, Saudi Arabia. J Infect Dev Ctries. 2022 Nov 29;16(11):1762–7.

14. Castro-Sánchez E, Moore LSP, Husson F, Holmes AH. What are the factors driving antimicrobial resistance? Perspectives from a public event in London, England. BMC Infect Dis. 2016 Dec;16(1):465.

15. Ocan M, Obuku EA, Bwanga F, Akena D, Richard S, Ogwal-Okeng J, et al. Household antimicrobial self-medication: a systematic review and meta-analysis of the burden, risk factors and outcomes in developing countries. BMC Public Health. 2015 Dec;15(1):742.

16. Shah J, Trautner BW, Olmeda K, Laytner LA, Faustinella F, Paasche-Orlow MK, et al. A survey of patient practices regarding leftover antibiotics reveals a potential source of antibiotic overuse. Tamma PD, editor. Antimicrob Agents Chemother. 2024 Aug 7;68(8):e00469-24.

17. Sachdev C, Anjankar A, Agrawal J. Self-Medication With Antibiotics: An Element Increasing Resistance. Cureus. 2022 Oct;14(10):e30844.

18. Machongo RB, Mipando ALN. "I don't hesitate to use the left-over antibiotics for my child" practices and experiences with antibiotic use among caregivers of pediatric patients at Zomba Central Hospital in Malawi. BMC Pediatr. 2022 Dec;22(1):466.

19. Social Determinant of Health [Internet]. Healthy people 2030; Available from: https://odphp.health.gov/healthypeople/priority-areas/social-determinants-health

20. Social determinants of health (WHO) [Internet]. World Health Organization (WHO); Available from: https://www.who.int/health-topics/social-determinants-of-health#tab=tab_1

21. Braveman P, Gottlieb L. The social determinants of health: it's time to consider the causes of the causes. Public Health Rep. 2014;129 Suppl 2(Suppl 2):19–31.

22. Breisinger C, Khouri N, Glauber JW, Laborde Debucquet D. One of the world's worst economic collapses, now compounded by the Ukraine crisis: What's next for Lebanon?

[Internet]. 0 ed. Washington, DC: International Food Policy Research Institute; 2023 [cited 2025 Jan 18]. Available from: https://hdl.handle.net/10568/140097

23. Bou Sanayeh E, El Chamieh C. The fragile healthcare system in Lebanon: sounding the alarm about its possible collapse. Health Econ Rev. 2023 Apr 4;13(1):21.

24. The World Bank in Lebanon [Internet]. 2022. Available from: https://www.worldbank.org/en/country/lebanon/overview

25. WHO. Lebanon: a conflict particularly destructive to health care [Internet]. 2024 Nov. Available from: https://www.who.int/news/item/22-11-2024-lebanon--a-conflict-particularly-destructive-to-health-care

26. Alhomoud F, Aljamea Z, Almahasnah R, Alkhalifah K, Basalelah L, Alhomoud FK. Self-medication and self-prescription with antibiotics in the Middle East—do they really happen? A systematic review of the prevalence, possible reasons, and outcomes. International Journal of Infectious Diseases. 2017 Apr;57:3–12.

27. Al-Mehmadi B, Alsubaie S, Al-Morikhi O, Alqahtani F, Almutairi W, Al-Mutairi M, et al. Knowledge and Attitude of self-medication with leftover antibiotics in Saudi Arabia: A cross-sectional study. F1000Res. 2024 Oct 18;12:304.

28. Bhat MJ, Al-qahtani M, Badawi AS, Asiri GB, Alhmare AM, Rashid A, et al. Awareness and Knowledge of Antibiotic Resistance and Risks of Self-Medication With Antibiotics Among the Aseer Region Population, Saudi Arabia, 2023. Cureus [Internet]. 2023 Jun 21 [cited 2024 Aug 5]; Available from: https://www.cureus.com/articles/164396-awareness-and-knowledge-of-antibiotic-resistance-and-risks-of-self-medication-with-antibiotics-among-the-aseer-region-population-saudi-arabia-2023

29. Henaine AM, Lahoud N, Abdo R, Shdeed R, Safwan J, Akel M, et al. Knowledge of Antibiotics Use among Lebanese Adults: A study on the influence of sociodemographic characteristics. Sultan Qaboos Univ Med J. 2021 Aug;21(3):442–9.

30. Jaber Chehayeb R, Kala S, Abou Ghannam H, Hasan G, Salloum J. The evolving Lebanese drug crisis: Trends in drug availability and affordability for common outpatient diseases from 2019 to 2023. Luyckx VA, editor. PLOS Glob Public Health. 2023 Nov 1;3(11):e0002538.

31. Mallah N, Orsini N, Figueiras A, Takkouche B. Income level and antibiotic misuse: a systematic review and dose-response meta-analysis. Eur J Health Econ. 2022 Aug;23(6):1015–35.

32. Norris P, Chamberlain K, Dew K, Gabe J, Hodgetts D, Madden H. Public Beliefs about Antibiotics, Infection and Resistance: A Qualitative Study. Antibiotics (Basel). 2013 Nov 5;2(4):465–76.

33. Rizk NA, Moghnieh R, Haddad N, Rebeiz MC, Zeenny RM, Hindy JR, et al. Challenges to Antimicrobial Stewardship in the Countries of the Arab League: Concerns of Worsening

Resistance during the COVID-19 Pandemic and Proposed Solutions. Antibiotics. 2021 Oct 29;10(11):1320.

34. Melki IS. Household crowding index: a correlate of socioeconomic status and inter-pregnancy spacing in an urban setting. Journal of Epidemiology & Community Health. 2004 Jun 1;58(6):476–80.

35. Al-Omrani H, Marwah MK, Al-Whaib R, Mekkawy M, Shokr H. Patterns of Drug Utilization and Self-Medication Practices: A Cross Sectional Study. Pharmacy (Basel). 2023 Nov 22;11(6):183.

36. Claire Marie-Armillo, April Jomerlynn-Salarda-Corsiga, Rina Mae-Gotam-Enriquez, Jim Denver-Libang, Juan Miguel-Paredes-Lucban, Marc Angelo-Matias-Roxas, et al. Level of self-medication awareness to internet usage among selected, non-medical students in Centro Escolar university-Manila during COVID-19 pandemic. GSC Biol Pharm Sci. 2022 Aug 30;20(2):152–63.

37. Sacre H, Haddad C, Hajj A, Zeenny RM, Akel M, Salameh P. Development and validation of the Socioeconomic Status Composite Scale (SES-C). BMC Public Health. 2023 Aug 24;23(1):1619.

38. Peek ME, Nunez-Smith M, Drum M, Lewis TT. Adapting the everyday discrimination scale to medical settings: reliability and validity testing in a sample of African American patients. Ethn Dis. 2011;21(4):502–9.

39. Mannasaheb BA, Alajlan SA, Alshahrani JA, Othman N, Alolayan SO, Alamrah MS, et al. Prevalence, Predictors and Point of View Toward Self-Medication Among Residents of Riyadh, Saudi Arabia: A Cross-Sectional Study. Front Public Health. 2022;10:862301.

40. Araia ZZ, Gebregziabher NK, Mesfun AB. Self-medication practice and associated factors among students of Asmara College of Health Sciences, Eritrea: a cross sectional study. J Pharm Policy Pract. 2019;12:3.

41. Al-Qerem W, Bargooth A, Jarab A, Akour A, Abu Heshmeh S. Information sources, attitudes, and practices of Self-Medication by Jordanian patients: A cross-sectional study. Saudi Pharmaceutical Journal. 2023 Apr;31(4):482–91.

42. Adhikari P. Perceptions And Practices Of Self-Medication Among The Residents Of Western, Nepal. J Manmohan Memorial Inst Health Sci. 2023 Nov 6;8(2):127–37.

43. Lee F, Suryohusodo AA. Knowledge, attitude, and practice assessment toward COVID-19 among communities in East Nusa Tenggara, Indonesia: A cross-sectional study. Front Public Health. 2022 Oct 31;10:957630.

44. Bianco A, Licata F, Zucco R, Papadopoli R, Pavia M. Knowledge and practices regarding antibiotics use. Evolution, Medicine, and Public Health. 2020 Jan 1;2020(1):129–38.

45. Sullman MJM, Lajunen TJ, Baddal B, Apostolou M. Antibiotics Knowledge, Attitudes and Behaviours among the Population Living in Cyprus. Antibiotics. 2023 May 12;12(5):897.

46. Sitotaw B, Philipos W. Knowledge, Attitude, and Practices (KAP) on Antibiotic Use and Disposal Ways in Sidama Region, Ethiopia: A Community-Based Cross-Sectional Survey. Mansour A, editor. The Scientific World Journal. 2023 Jul 26;2023:1–15.

47. Wong LP, Alias H, Husin SA, Ali ZB, Sim B, Ponnampalavanar SSLS. Factors influencing inappropriate use of antibiotics: Findings from a nationwide survey of the general public in Malaysia. Shankar PR, editor. PLoS ONE. 2021 Oct 20;16(10):e0258698.

48. Kosiyaporn H, Chanvatik S, Issaramalai T, Kaewkhankhaeng W, Kulthanmanusorn A, Saengruang N, et al. Surveys of knowledge and awareness of antibiotic use and antimicrobial resistance in the general population: A systematic review. Tu WJ, editor. PLoS ONE. 2020 Jan 16;15(1):e0227973.

49. Ansari M, Alanazi A, Moin A. Consumers' awareness, attitude and associated factors towards self-medication in Hail, Saudi Arabia. PLoS One. 2020;15(4):e0232322.

50. Charlie Parker, Sam Scott, Alistair Geddes. Parker, C, Scott, S and Geddes, A (2019) Snowball Sampling. SAGE Research Methods Foundations. 2019; Available from: https://core.ac.uk/download/pdf/211022791.pdf

51. Goodyear MDE, Krleza-Jeric K, Lemmens T. The Declaration of Helsinki. BMJ. 2007 Sep 29;335(7621):624–5.

52. El Baba J. Roots of Lebanon's Sectarian Politics: Colonial Legacies of the French Mandate. IAPSS Politikon. 2024 Nov 30;58:26–49.

53. Chatty D, Mansour N, Yassin N. Bedouin in Lebanon: Social discrimination, political exclusion, and compromised health care. Social Science & Medicine. 2013 Apr;82:43–50.

54. Cammett M. Sectarianism and the Ambiguities of Welfare in Lebanon. Current Anthropology. 2015 Oct;56(S11):S76–87.

55. Awada S, Diab B, Khachman D, Zeidan RK, Slim H, Zein S, et al. Self-medication Practices and Knowledge among Lebanese Population: A Cross-sectional Study: DSAHMJ. 2020;2(2):56.

56. Mallah N, Badro DA, Figueiras A, Takkouche B. Association of knowledge and beliefs with the misuse of antibiotics in parents: A study in Beirut (Lebanon). Perez SF, editor. PLoS ONE. 2020 Jul 22;15(7):e0232464.

57. Pandey AK, Cohn J, Nampoothiri V, Gadde U, Ghataure A, Kakkar AK, et al. A systematic review of antibiotic drug shortages and the strategies employed for managing these shortages. Clinical Microbiology and Infection. 2024 Sep;S1198743X24004555.

58. Richards AR, Linder JA. Behavioral Economics and Ambulatory Antibiotic Stewardship: A Narrative Review. Clin Ther. 2021 Oct;43(10):1654–67.

59. Abdi A, Faraji A, Dehghan F, Khatony A. Prevalence of self-medication practice among health sciences students in Kermanshah, Iran. BMC Pharmacol Toxicol. 2018 Dec;19(1):36.

60. Tomas Petrović A, Pavlović N, Stilinović N, Lalović N, Paut Kusturica M, Dugandžija T, et al. Self-Medication Perceptions and Practice of Medical and Pharmacy Students in Serbia. IJERPH. 2022 Jan 21;19(3):1193.

61. Haraoui LP, Rizk A, Landecker H. States of Resistance: nosocomial and environmental approaches to antimicrobial resistance in Lebanon. HPLS. 2024 Sep;46(3):28.

62. World Health Organization (WHO). Antimicrobial resistance: global report on surveillance [Internet]. Available from: https://www.who.int/publications/i/item/9789241564748

63. Sun R, Yao T, Zhou X, Harbarth S, Lin L. Non-biomedical factors affecting antibiotic use in the community: a mixed-methods systematic review and meta-analysis. Clinical Microbiology and Infection. 2022 Mar;28(3):345–54.

64. Barker AK, Brown K, Ahsan M, Sengupta S, Safdar N. Social determinants of antibiotic misuse: a qualitative study of community members in Haryana, India. BMC Public Health. 2017 Dec;17(1):333.

65. Connell CL, Wang SC, Crook L, Yadrick K. Barriers to Healthcare Seeking and Provision Among African American Adults in the Rural Mississippi Delta Region: Community and Provider Perspectives. J Community Health. 2019 Aug;44(4):636–45.

66. Vedder A, Wachbroit R. Reliability of information on the Internet: Some distinctions. Ethics and Information Technology. 2003;5(4):211–5.

67. Chen J, Tian Y, Yin M, Lin W, Tuersun Y, Li L, et al. Relationship between self-efficacy and adherence to self-management and medication among patients with chronic diseases in China: A multicentre cross-sectional study. Journal of Psychosomatic Research. 2023 Jan;164:111105.

68. de Hoog N, van Dinther S, Bakker E. Socioeconomic Status and Health-Compromising Behaviour: Is it All About Perception? Eur J Psychol. 2020 Aug;16(3):498–513.

69. Pampel FC, Krueger PM, Denney JT. Socioeconomic Disparities in Health Behaviors. Annu Rev Sociol. 2010 Aug;36:349–70.

70. Doty MM, Tikkanen RS, FitzGerald M, Fields K, Williams RD. Income-Related Inequality In Affordability And Access To Primary Care In Eleven High-Income Countries: Study reports survey results on health status, socioeconomic risk factors, affordability, and access to primary care among adults in the US and ten other high-income countries. Health Affairs. 2021 Jan 1;40(1):113–20.

71. Singh-Phulgenda S, Antoniou P, Wong DLF, Iwamoto K, Kandelaki K. Knowledge, attitudes and behaviors on antimicrobial resistance among general public across 14 member states in the WHO European region: results from a cross-sectional survey. Front Public Health. 2023;11:1274818.

72. Khaddaj R. Data Collection (Social Determinant of LA use).xlsx [Internet]. figshare; 2025 [cited 2025 Feb 23]. p. 240468 Bytes. Available from:

https://figshare.com/articles/dataset/Data_Collection_Social_Determinant_of_LA_use_xlsx/28465301

Figure 1

Click here to access/download;Figure;image.png

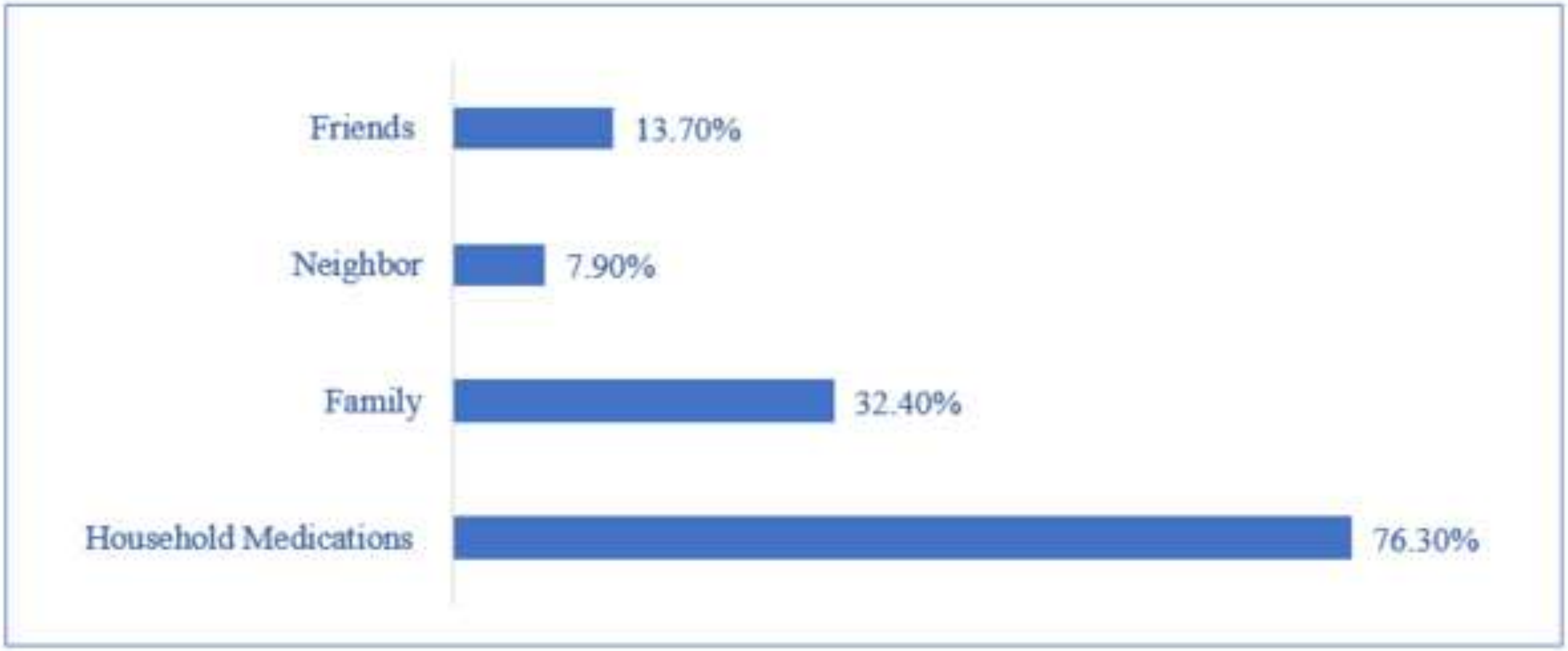

Figure 1: Source of Leftover Antibiotics Use

Figure 2

Click here to access/download;Figure;Screenshot 2025-02-28 150025.png

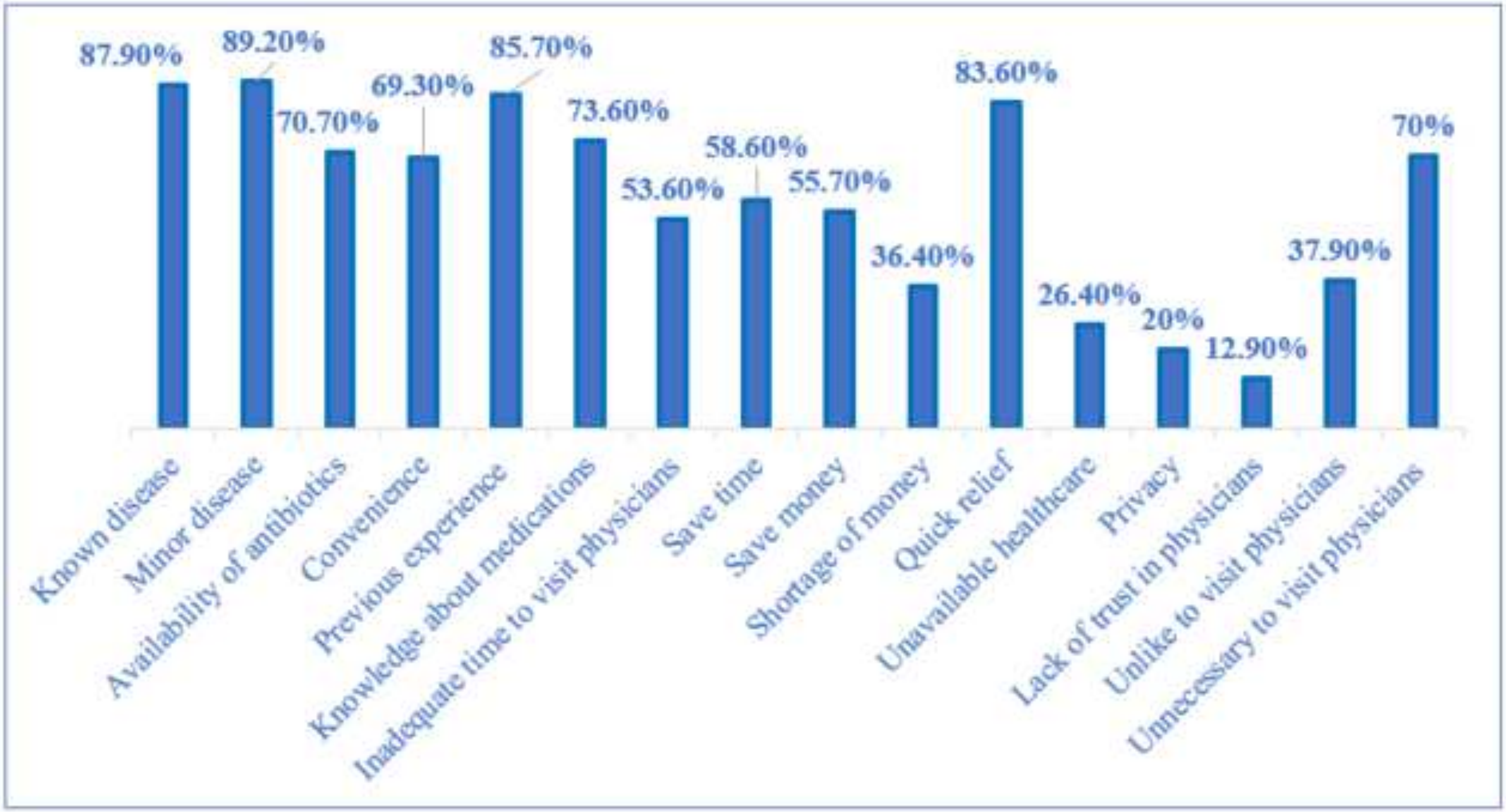

Figure 2: Reasons Encouraging Self-Medication with Antibiotic Leftover

Figure 3 Click here to access/download;Figure;Screenshot 2025-02-28 150132.png

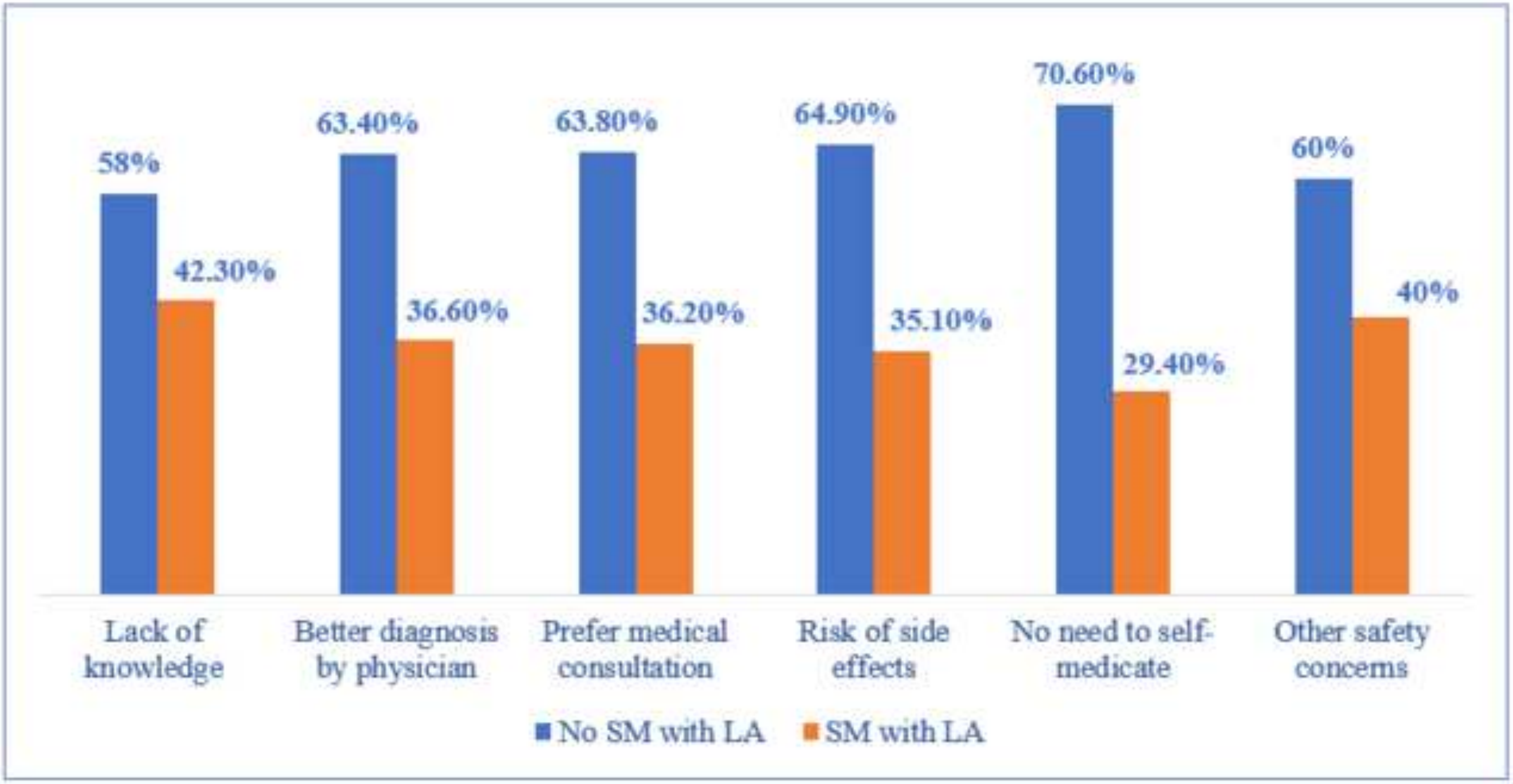

Figure 3: Reasons for avoiding leftover antibiotic use among self-medicating and non-self-medicating participants.
