# Supplementary material for: Social determinants of self-medication with leftover antibiotics in Lebanese households: A cross-sectional study
Source: PLoS One. 2025 Sep 25;20(9):e0333377. doi: 10.1371/journal.pone.0333377 (PMC12463266; doi:10.1371/journal.pone.0333377)
Supplement: Attachment — Submitted filename: Response to Reviewers.docx [file pone.0333377.s004.docx]

Dear reviewers and editors,

I would like to sincerely thank you for the time and effort you dedicated to reviewing my manuscript entitled “Social Determinants of Self-Medication with Leftover Antibiotics in Lebanese Households: A Cross-Sectional Study.”

Below, we provide a detailed response to each reviewer’s question. We have carefully addressed all the concerns raised.

1- Please ensure that your manuscript meets PLOS ONE's style requirements, including those for file naming: Checked and corrected
2- Please provide additional details regarding participant consent: Written informed consent was obtained from all participants by including a consent statement at the beginning of the online survey. Participants were required to read the statement and click a checkbox indicating their agreement to participate, which enabled them to proceed
3- Please include a complete copy of PLOS’s questionnaire on inclusivity in global research in your revised manuscript: Questionnaire uploaded in the requested format.
4- Please ensure that you refer to Figure 1-3 in your text: this was already done, and the figures are separately submitted as per the journal guidelines
5- The question about: how was health education measured? Health education was measured using two questions in the survey. The first question asked about the participant’s level of education. The second question asked if they had studied in a health-related field, such as pharmacy, medicine, nursing, or public health. This helped us identify participants with a background in health.
6- The question about: How does drug shortage fit in socio-economic and political conditions? The socioeconomic crisis and the political instability in Lebanon were the main causes for the drug shortage. Many references support this:
   a. *Fuel paralysis: How 14 private companies control Lebanon’s oil supply and set its prices [Internet]. thecradle.co. Available from:* [*https://thecradle.co/articles-id/5690*](https://thecradle.co/articles-id/5690)
   b. *Frantic search after medicines vanish from Lebanon shelves | AP News [Internet]. AP News. 2020. Available from:* [*https://apnews.com/article/lebanon-medicines-vanish-from-shelves-dbf0319ed8a4f248fb0fb3040c371fdb*](https://apnews.com/article/lebanon-medicines-vanish-from-shelves-dbf0319ed8a4f248fb0fb3040c371fdb)
   c. *Jaber Chehayeb R, Kala S, Abou Ghannam H, Hasan G, Salloum J. The evolving Lebanese drug crisis: Trends in drug availability and affordability for common outpatient diseases from 2019 to 2023. Luyckx VA, editor. PL*
7- The question about: if all Lebanese people speak English, and about the forward and backward translation: Not all Lebanese people speak English; for this reason, the questionnaire was initially prepared in English, and then it was translated into Arabic. To ensure adequate translation, a forward translation (English to Arabic) was initially performed, followed by a back translation (Arabic to English).
8- The question about the p-value: Why use variables with p-value <0.2 for the logistic regression? A common threshold for statistical significance is 0.05. If the p-value is below this level, the relationship is considered statistically significant. However, p-values between 0.05 and 0.2 are often considered marginally significant, suggesting a possible association that may not meet conventional significance levels but still warrants attention. In such cases, a variable may still be relevant, especially if there is a strong theoretical or contextual justification for its inclusion. Variables with marginal p-values may reflect important real-world influences and can be included in the model to support interpretation and guide future research. And here is a reference for this: *Zhang Z. Model building strategy for logistic regression: purposeful selection. Ann Transl Med. 2016 Mar;4(6):111–111.*
9- The question related to which statistical tests were used? As mentioned in the methods. For categorical variables (education, chronic disease, gender…), Chi-square tests were used. Whereas for continuous variables (Age and HCI), the Student’s t-test was applied.
10- The question asking if I mean the perception of the political conflict. My answer is YES, the political and economic instability in Lebanon and the region has affected their perception regarding medication use and storage.
11- The question asking for details regarding the issues in prescription was detailed in the part related to this.
12- The question related to a more robust discussion related to family borrowing patterns has been elaborated in the part related to this.
13- The question about the equalizing effect: The political instability and ongoing socioeconomic crisis in Lebanon have affected the entire population, regardless of their economic status. Whether wealthy or poor, people across all social and financial backgrounds have experienced the consequences: from rising prices and inflation to shortages of essential goods and services. Even those who were previously financially secure have faced difficulties as the crisis has disrupted everyday life, reduced purchasing power, and created uncertainty about the future. This widespread impact highlights how deeply the crisis permeates all layers of society, leaving no one untouched.
14- For the question stating that there is a blanket statement: *The relationship between discrimination and LA use reveals a unique dynamic within Lebanon's sectarian healthcare system.* This statement is explained directly after: LA users reported lower scores on the discrimination scale, suggesting that reduced discrimination correlates with greater access to medications.
15- For the question asking for a reference regarding the healthcare access based on the socio-economic situation in Lebanon: I added one article to the discussion part, but there is also an e-book published by the Ministry of Public Health in Lebanon entitled “Health Beyond Politics-2009” that can serve as an important reference also. It can be found on the following link: [*https://www.moph.gov.lb/en/view/3908/health-beyond-politics*](https://www.moph.gov.lb/en/view/3908/health-beyond-politics)
16- Regarding the question related to elaborating on the crisis in Lebanon: This is already mentioned in the introduction. I will be elaborating on it more.

We sincerely thank the reviewers and editors for their valuable comments and constructive feedback, which have greatly contributed to improving the quality and clarity of our manuscript.

We hope that the revised version adequately addresses all concerns, and we remain open to any further suggestions.
